# Rhesus Theta Defensin 1 Promotes Long Term Survival in Systemic Candidiasis by Host Directed Mechanisms

**DOI:** 10.1038/s41598-019-53402-z

**Published:** 2019-11-15

**Authors:** Virginia Basso, Dat Q. Tran, Justin B. Schaal, Patti Tran, Yoshihiro Eriguchi, Diana Ngole, Anthony E. Cabebe, A. young Park, Paul M. Beringer, André J. Ouellette, Michael E. Selsted

**Affiliations:** 1 Department of Pathology and Laboratory Medicine, Keck School of Medicine, University of Southern California, Los Angeles, California United States of America; 2 Oryn Therapeutics, Vacaville, California United States of America; 3 Department of Clinical Pharmacy, School of Pharmacy, University of Southern California, Los Angeles, United States of America; 4 Norris Comprehensive Cancer Center of the University of Southern California, Los Angeles, California United States of America; 5 Present Address: Department of Clinical Immunology and Rheumatology/Infectious DiseaseKyushu University HospitalDepartment of Medicine and Biosystemic ScienceKyushu University Graduate School of Medical Science, Fukuoka, Japan

**Keywords:** Immunology, Microbiology

## Abstract

Invasive candidiasis is an increasingly frequent cause of serious and often fatal infections in hospitalized and immunosuppressed patients. Mortality rates associated with these infections have risen sharply due to the emergence of multidrug resistant (MDR) strains of *C*. *albicans* and other *Candida* spp., highlighting the urgent need of new antifungal therapies. Rhesus theta (θ) defensin-1 (RTD-1), a natural macrocyclic antimicrobial peptide, was recently shown to be rapidly fungicidal against clinical isolates of MDR *C*. *albicans in vitro*. Here we found that RTD-1 was rapidly fungicidal against blastospores of fluconazole/caspofungin resistant *C*. *albicans* strains, and was active against established *C*. *albicans* biofilms *in vitro*. *In vivo*, systemic administration of RTD-1, initiated at the time of infection or 24 h post-infection, promoted long term survival in candidemic mice whether infected with drug-sensitive or MDR strains of *C*. *albicans*. RTD-1 induced an early (4 h post treatment) increase in neutrophils in naive and infected mice. *In vivo* efficacy was associated with fungal clearance, restoration of dysregulated inflammatory cytokines including TNF-α, IL-1β, IL-6, IL-10, and IL-17, and homeostatic reduction in numbers of circulating neutrophils and monocytes. Because these effects occurred using peptide doses that produced maximal plasma concentrations (Cmax) of less than 1% of RTD-1 levels required for *in vitro* antifungal activity in 50% mouse serum, while inducing a transient neutrophilia, we suggest that RTD-1 mediates its antifungal effects *in vivo* by host directed mechanisms rather than direct fungicidal activity. Results of this study suggest that θ-defensins represent a new class of host-directed compounds for treatment of disseminated candidiasis.

## Introduction

Systemic infection caused by MDR fungi is a growing global health concern. It is estimated that approximately 1.5 million cases of disseminated mycoses occur annually and are associated with high mortality rates^1–3^. Fungal pathogens are a major cause of hospital-acquired infection^4^, particularly among surgical patients and those with indwelling catheters^5^. Increased risk of systemic fungal infection is also associated with biologic therapies for treatment of inflammatory or autoimmune diseases^6,7^.

Among fungal infections, *Candida* spp. are the most frequent causative organisms worldwide^4,8^. Over 400,000 cases of candidiasis occur annually^9^ with increased risk of infection associated with defects in innate immunity, neutropenia, and diabetes^10–12^. The growing incidence of MDR *Candida* spp. infections has contributed to the increase in mortality rates associated with systemic candidiasis^13–15^. A major risk factor for systemic candidiasis is the presence of biofilms that commonly colonize implanted medical devices such as venous catheters. Biofilms are notoriously resistant to antifungal therapy and are the source of blood borne dissemination^16–19^.

The failure to develop agents that are selective against eukaryotic pathogens has impeded the clinical introduction of new antifungals^14^. Currently, there are but three classes of antifungal drugs that are relied upon for treatment of invasive fungal infections: polyenes, azoles, and echinocandins^20^. Echinocandins, introduced nearly 20 years ago, are the most recently approved class of antifungals. Limitations associated with use of currently available agents include limited activity spectra^17,21,22^, serious adverse side effects^23,24^, and lack of activity against biofilms^25,26^. The emergence of MDR fungal pathogens underscores the urgent need for development of new approaches for treatment of fungal infections^27–29^.

θ-defensins are macrocyclic peptides containing an 18 amino acid cyclic backbone stabilized by a tri-disulfide core^30^. Numerous naturally occurring θ-defensin isoforms are expressed in Old World monkeys (OWM) such as macaques, baboons, and vervets^31–34^, but the peptides are absent in New World monkeys, apes and humans^30^. RTD-1, the prototype rhesus macaque θ-defensin, is effective in preclinical models of polymicrobial and *E*. *coli* sepsis^35^, SARS-coronavirus infection^36^, a mouse model of *P*. *aeruginosa* induced cystic fibrosis^37^, and endotoxin-induced lung injury^38^. In a recent study we reported that θ-defensins are potently fungicidal *in vitro* against MDR *C*. *albicans* and non-albicans *Candida* spp., including the emerging pathogen *Candida auris*^39^.

In the current study we evaluated the antifungal activities of RTD-1 against planktonic cells and biofilms of drug sensitive and MDR *C*. *albicans* strains *in vitro*, and found the peptide to be fungicidal against both forms of each strain. We then tested RTD-1 for efficacy in a therapeutic mouse model of systemic candidiasis and analyzed the effects of peptide treatment on survival, fungal clearance, and on inflammatory biomarkers in infections mediated by drug-sensitive and MDR isolates of *C*. *albicans*.

## Results

### RTD-1 is fungicidal against blastospores of MDR *C*. *albicans*

Based on the finding that RTD-1 is potently and rapidly fungicidal against several *C*. *albicans* and non-albicans *Candida* spp., including MDR isolates^39^, we tested for activity of the peptide against blastospores of two caspofungin (Caspo)-resistant *C*. *albicans* clinical isolates 43001 and 53264^40^, as well as the genetically defined, drug-sensitive reference strain SC5314. Minimum inhibitory concentration (MICs) and minimal fungicidal concentration (MFCs) determinations demonstrated that the Caspo-resistant clinical isolates were resistant to fluconazole (Fluco), and were 33-fold (strain 43001) or >133-fold (strain 53264) more resistant to Caspo than drug sensitive SC5314 (Table 1) consistent with a previous study^40^. RTD-1 was fungicidal against all three strains with MICs/MFCs ranging from 6.25–25 µg/ml (Table 1), and MFCs were 1–2 × the MICs (Table 1). RTD-1 was 5 to >40 times more active than Fluco against the three strains tested under standard conditions (Table 1).

**Table 1:** Minimum Inhibitory Concentration (MIC) and Minimum Fungicidal Concentration (MFC) of θ-defensin RTD-1 and clinical antifungals^a^.

	RPMI						50% serum	
*C*. *albicans* #	RTD-1		Fluco		Caspo		RTD-1	
	MIC	MFC	MIC	MFC	MIC	MFC	MIC	MFC
SC5314	12.5	25	64	>256	0.06	0.25	>100	>100
43001	6.25	12.5	>256	>256	2	2	>100	>100
53264	12.5	12.5	>256	>256	>8	>8	>100	>100

^a^A modified CLSI protocol, described in Methods, was used to determine MICs for RTD-1, fluconazole (Fluco) and caspofungin (Caspo). MFC assays were conducted as described in Material and Methods. MFC values correspond to 99% killing relative to input inoculum. MICs and MFCs for RTD-1 were determined also in 50% serum as described in Material and Methods.

**Figure 1:**
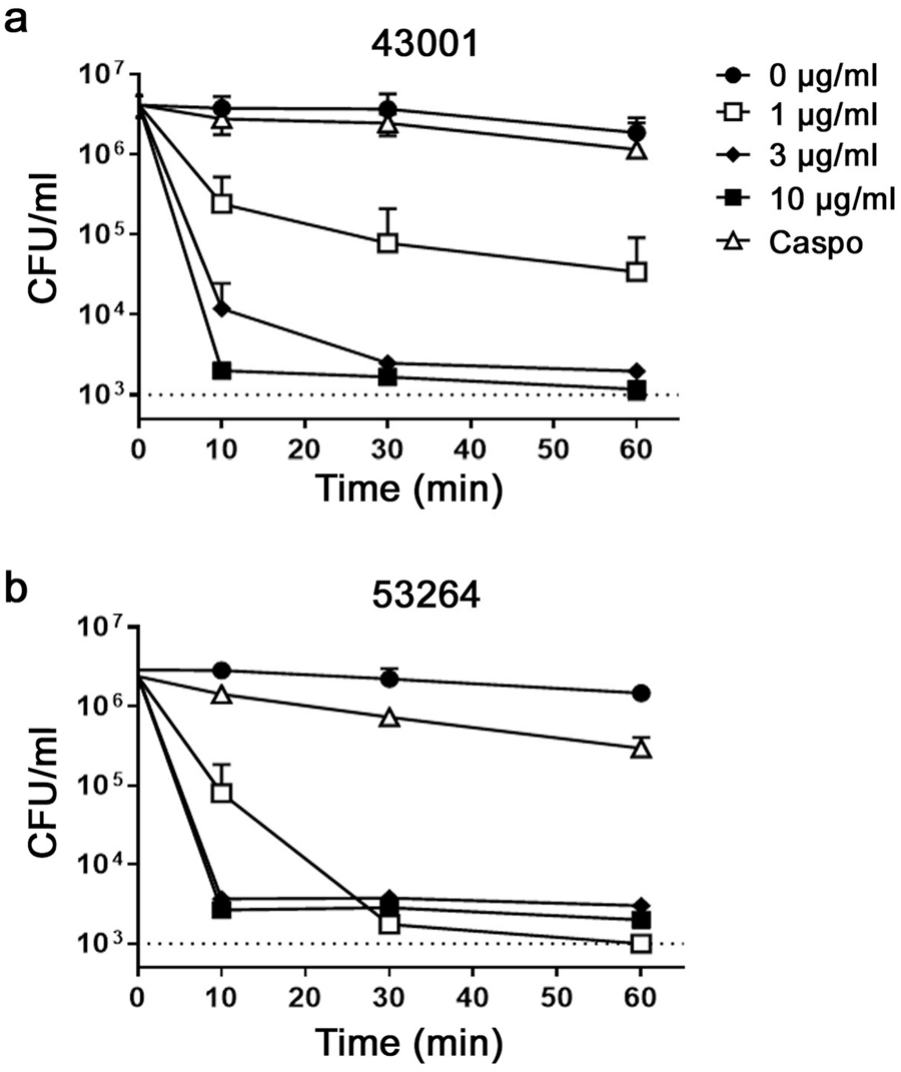
Concentration and time dependence of RTD-1 fungicidal activity. *C*. *albicans* clinical isolates 43001 (**a**) and 53264 (**b**) were incubated with indicated concentrations of RTD-1 or caspofungin (10 µg/ml) in PIPES-glucose buffer for 0–60 min. Data, plotted as mean CFU/ml +/− S.D, and are representative of experiments performed in triplicate.

As previously reported, drug sensitive *C*. *albicans* SC5314 and MDR *C*. *albicans* strains are rapidly and concentration-dependently killed by RTD-1 and related θ-defensin isoforms^39^. RTD-1 showed similar time and concentration-dependent killing of Caspo-resistant 43001 and 53264, with peptide concentrations as low as 1 µg/ml killing >90% of input blastospores within 10 min of peptide exposure (Fig. 1), and 3 µg/ml sterilized the incubation mixture by 30 min of incubation. Under these conditions, 10 µg/ml Caspo showed very little activity against either strain (Fig. 1).

Antifungal activity of RTD-1 against fungal blastospores was also evaluated in the presence of 50% mouse serum. In the presence of serum, RTD-1 was inactive against *C*. *albicans* SC5314, 43001 and 53264 at peptide concentrations up to 100 µg/ml (Table 1). The significance of this finding is discussed further below.

### RTD-1 is active against *C*. *albicans* biofilms

**Figure 2:**
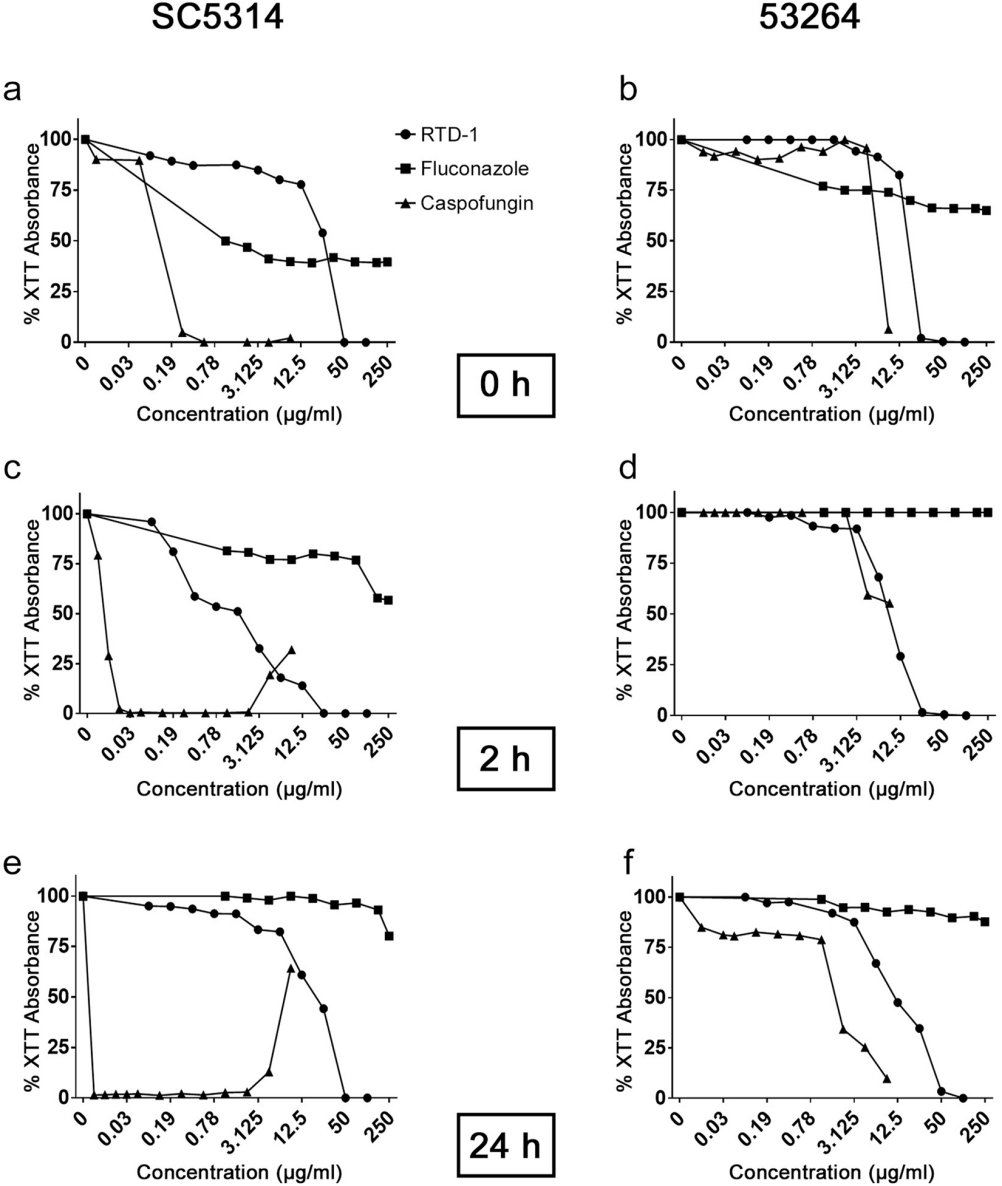
RTD-1 efficacy against biofilms. RTD-1 was tested for the ability to prevent biofilm formation of *C*. *albicans* SC5314 and 53264 (**a**,**b**), formation of biofilms after 2 h of adhesion (**c**,**d**), and disruption of mature biofilms (**e**,**f**). In each condition, biofilm metabolism was evaluated by XTT absorbance 24–48 h after addition of the indicated concentration of RTD-1, fluconazole or caspofungin. The highest concentrations tested were 100 μg/ml (48 μM) RTD-1, 250 μg/ml (816.3 μM) fluconazole (Fluco), and 8 μg/ml (7.3 μM) caspofungin (Caspo).

Since fungal biofilms are generally resistant to conventional antifungals, we analyzed the activity of RTD-1, Fluco and Caspo against biofilms of *C*. *albicans* SC5314 and MDR isolate 53264. Each agent was tested for its effect on biofilm formation by incubating fungal cell preparations with each agent prior to adhesion, 2 h after adhesion to plastic, or after mature (24 h) biofilms were established (Fig. 2). RTD-1 inhibited biofilms of drug sensitive SC5314 at each time point, but less effectively than Caspo. Under pre-adhesion conditions, Fluco was more effective than RTD-1 at concentrations below 50 µg/ml. However, RTD-1 was much more effective than Fluco in the 2- and 24 h assays (Fig. 2). Of note, the antifungal effect of Caspo against 2- and 24 h SC5314 biofilms was reduced at concentrations >2 µg/ml, consistent with the so-called “paradoxical” or “eagle” effect previously described^41,42^.

RTD-1 also inhibited biofilm formation of MDR strain 53264. Unlike SC5314, 53264 was highly resistant to Fluco under each condition tested. Concentration-dependent 53264 biofilm inhibition by RTD-1 was similar to that of Caspo (Fig. 2). These data demonstrate that RTD-1 is effective against fungal biofilms *in vitro* with activity approximating that of Caspo against *C*. *albicans* 53264, an organism that is Fluco- (this study) and Caspo-resistant^40^.

### Systemic RTD-1 administration enhances survival in systemic candidiasis

**Figure 3:**
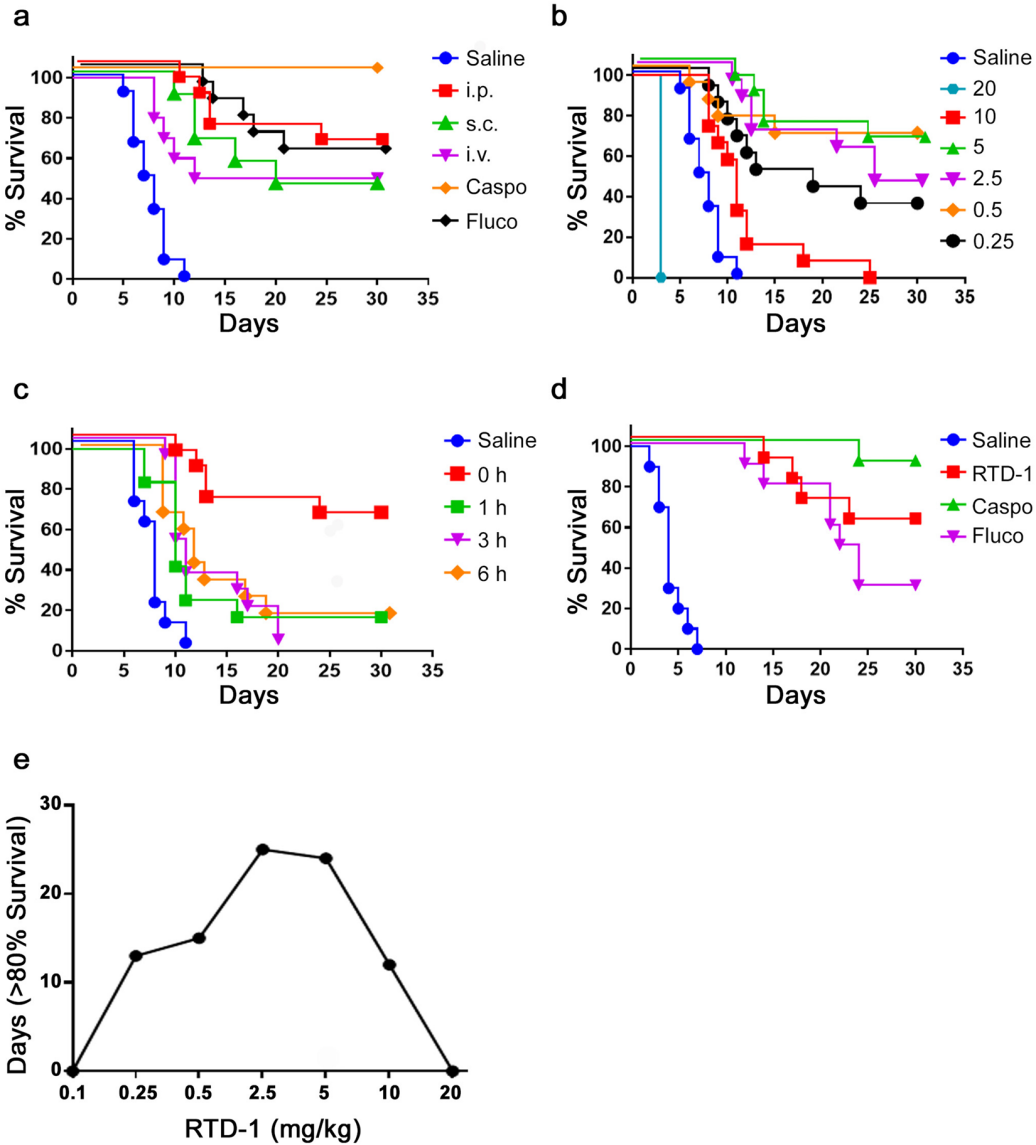
RTD-1 promotes survival of mice with disseminated *C*. *albicans* infection. (**a**) RTD-1 efficacy is independent of route of peptide administration. Mice infected i.v. with 3 × 10^5^ *C*. *albicans* SC5314 were treated once at T = 0 with 5 mg/kg RTD-1 administered i.p. (n = 13), i.v. (n = 12), or s.c. (n = 12). Cohorts receiving fluconazole (Fluco; n = 12) or caspofungin (Caspo; n = 12) were treated once, 5 mg/kg, by i.p. injection at T = 0. Controls received saline (n = 12) i.p at T = 0, and mice were monitored for 30 days. Relative to saline controls, RTD-1 treated mice had increased survival (*P* = 3.3 × 10^−7^, i.p.; *P* = 1.5 × 10^−5^, s.c.; *P* = 6.9 × 10^−4^, i.v.) as did Caspo and Fluco treated animals (*P* = 4.3 × 10^−7^). There was no statistical difference observed among groups treated with RTD-1, or between RTD-1 and Fluco, whereas Caspo was significantly more effective than RTD-1 or Fluco (see Suppl. Table 1). (**b**) Dose dependent efficacy of RTD-1. Mice (n = 12 or 13 for each cohort) were infected i.v. with SC5314 (as in panel **a**) and treated with a single i.p. dose (mg/kg shown) of RTD-1 at T = 0 and monitored for 30 d. RTD-1 significantly enhanced survival (*P* < 0.00001 for all treatment cohorts but the 10 mg/kg cohort for which *P* = 0.0013, and the 20 mg/kg for which RTD-1 significantly accelerates death, *P* = 6.3 × 10^−5^; see Suppl. Table 1 for statistical summary). (**c**) Effect of infection-treatment interval on single dose RTD-1 efficacy. Mice were infected i.v. (as in panel **a**) and treated i.p. with 5 mg/kg RTD-1 at intervals following infection: T = 0 (n = 13, data redrawn from Fig. panel **a**), 1 h (n = 12), 3 h (n = 12), and 6 h (n = 12). Saline (control) vehicle was administered i.p. at T = 0 and all mice were monitored for up to 30 days. While peptide treatment enhanced survival compared to saline control (*P* < 0.002 for all peptide treated cohort; Suppl. Table 1), delaying treatment reduced single dose efficacy of RTD-1. (**d**) Multiple RTD-1 dosing is effective in delayed treatment of systemic candidiasis. Mice (n = 10 for each group) were infected i.v. with *C*. *albicans* SC5314. Beginning 24 h p.i., mice received daily i.p. injections of 5 mg/kg of RTD-1, 5 mg/kg Fluco, 5 mg/kg Caspo, or saline for 7 days and mice were monitored for up to 30 d. All three agents markedly improved survival (*P* = 3.4 × 10^−6^) compared to saline treatment. Efficacy of RTD-1 was not statistically different than that of Fluco or Caspo, but Caspo was superior to Fluco (*P* = 4.7 × 10^−3^, Suppl. Table 1). (**e**) Inverted “U” effects of RTD-1 treatment of systemic candidiasis. Survival rates from Fig. 3b and RTD-1 0.1 mg/kg (not shown) are plotted as number of days showing >80% survival in function of RTD-1 concentration ranging from 0.1 to 20 mg/kg. RTD-1 enhanced survival as dose levels are increased up to 5 mg/kg, but decreased in efficacy with dosing at 10 and 20 mg/kg, resembles an inverted “U” shape.

The efficacy of RTD-1 *in vivo* was evaluated in an established model of systemic candidiasis using *C*. *albicans* SC5314. Both immunocompetent and immunosuppressed mouse models are employed in preclinical studies of systemic candidiasis. Since many of our preliminary studies employed the latter model, we conducted efficacy studies with RTD-1 and reference antifungal drugs in immunocompetent BALB/c mice. Mice were challenged i.v. with 3 × 10^5^ blastospores at T = 0, which rapidly produces disseminated candidiasis and colonizes major organs in a manner resembling systemic candidiasis in humans^43^. Under these conditions, SC5314 typically caused 100% terminal morbidity by day 12 in saline treated controls. We analyzed the effect of a single 5 mg/kg dose of RTD-1 administered i.v., s.c., or i.p. immediately after i.v. fungal challenge, with i.p. saline as vehicle control. Fluco and Caspo, administered i.p. at 5 mg/kg, were used as drug comparators. All saline treated SC5314 challenged mice died or became terminally moribund (requiring euthanasia) in 7–12 days, whereas single dose treatments with Caspo (*P* < 0.0001) or Fluco (*P* < 0.0001) were both effective in enhancing survival (Fig. 3a). A single dose of RTD-1 significantly enhanced survival regardless of the routes of administration (*P* < 0.0001 for i.p. and s.c., and *P* = 0.0007 for i.v.) and were not significantly different from each other, nor from Fluco treatment (Fig. 3a). Caspo treatment was superior to RTD-1 and Fluco treatment (*P ≤ *0.01 in each case; Fig. 3a). In subsequent studies, antifungal agents were administered i.p. to circumvent potentially confounding effects resulting from treatment by the same route as infection, which was i.v. in all experiments.

RTD-1 tolerability/toxicity was evaluated by i.p. dosing of naÏve BALB/c mice with RTD-1 at 5 mg/kg RTD-1 daily for 7 days, 20 mg/kg every other day for 10 days, and once at 50 mg/kg. Mice tolerated RTD-1 dosing at all levels, and all were clinically normal for observation periods of at least 14 days following the last escalating dose.

We next evaluated the administration of single i.p. doses of RTD-1 ranging from 0.1 to 20 mg/kg immediately after i.v. fungal challenge. With the exception of the lowest (0.1 mg/kg; not shown) and the highest dose tested (20 mg/kg, Fig. 3b), RTD-1 treatment enhanced survival significantly (*P* < 0.002) compared to saline controls (Fig. 3b). Enhanced survival curves following single doses of RTD-1 at 0.25, 0.5, 2.5, and 5.0 mg/kg were statistically equivalent to each other (Fig. 3b) and to administration with 5 mg/kg of Fluco (Fig. 3a,b). Interestingly, dosing with 10 and 20 mg/kg RTD-1 was less effective than Fluco (*P* < 0.0001) and to RTD-1 doses between 0.25 and 5.0 mg/kg (*P* ≤ 0.02 in all cases; see Suppl. Table 1).

We then analyzed the effect of delaying RTD-1 treatment following fungal challenge. Compared to RTD-1 dosing at the time of i.v. fungal challenge, a one hour delay reduced survival benefit (Fig. 3c). Nevertheless, even with treatment delays of 1, 3 or 6 h, a single i.p. dose of 5 mg/kg RTD-1 resulted in enhanced survival compared to saline control (*P* ≤ 0.002; Fig. 3c). However, no survival enhancement was achieved if peptide administration was delayed for 24 h or 72 h (data not shown).

We then determined whether multiple dose administration of RTD-1 would improve survival in mice in the delayed treatment format. Mice challenged i.v. with *C*. *albicans* SC5314 were treated i.p. once a day for 7 days with 5 mg/kg RTD-1, Caspo, or Fluco with the first dose of each agent given 24 h after fungal infection. Each agent markedly enhanced survival (*P* < 0.0002; Fig. 3d), but RTD-1 was more effective in enhancing survival than Fluco, and was nearly equivalent to Caspo. Body weights of long term survivors (≥25 days) of each treatment cohort did not differ statistically from those of uninfected mice 25–30 days post infection and were otherwise clinically normal (Fig. 3 and data not shown).

### Pharmacokinetics (PK) of systemically administered RTD-1

**Figure 4:**
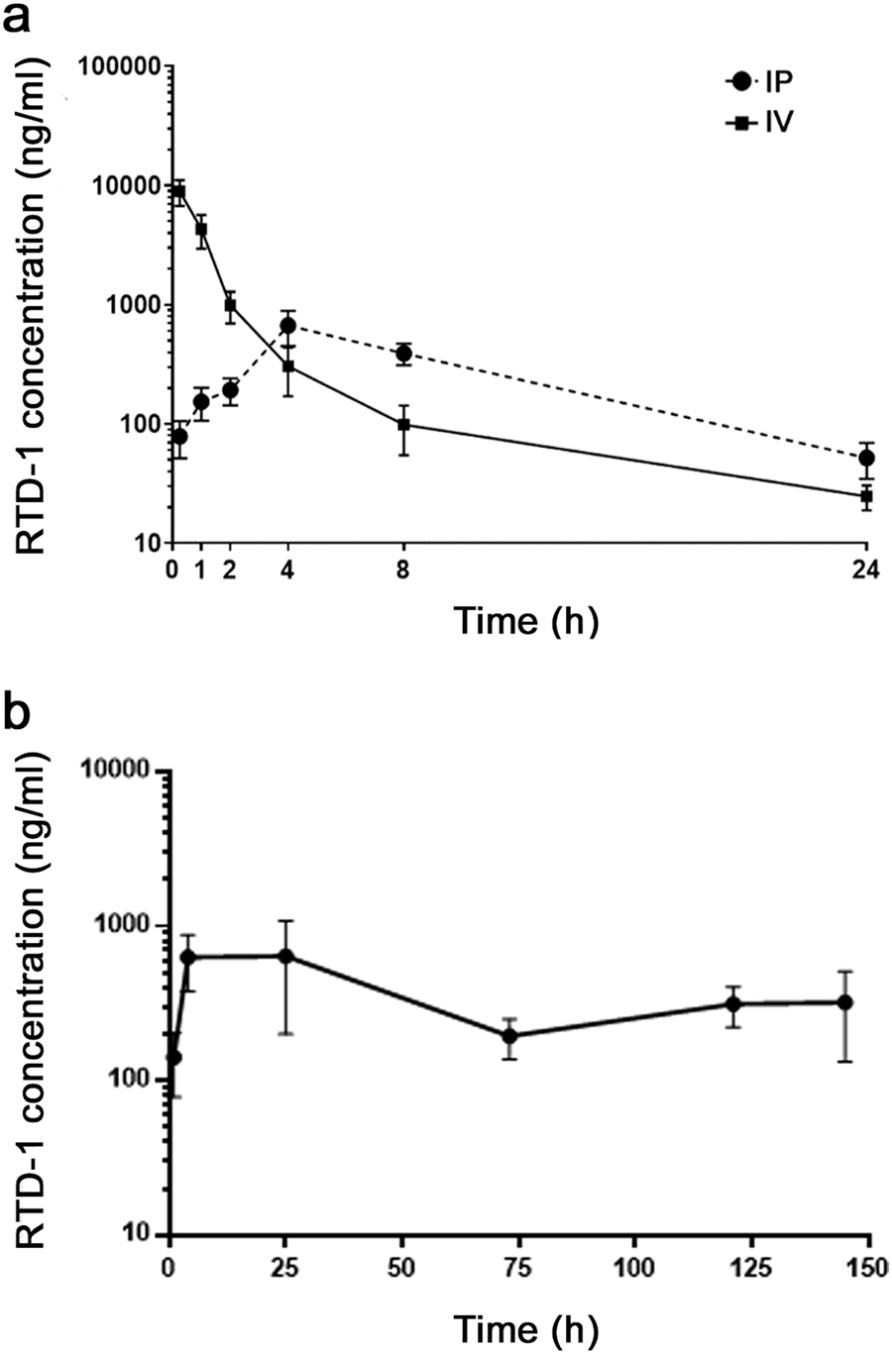
RTD-1 concentration-time profiles following i.v. and i.p. administration. (**a**) Single. dose PK. Single doses of RTD-1 (5 mg/kg) were administered i.v. or i.p. at T = 0 as described in Methods. Blood was collected at the indicated time points and plasma RTD-1 levels were quantified by LC-MS/MS. Mean plasma levels and SD are plotted at each time point. (**b**) Multiple dose administration of RTD-1. Mice received 1-7 doses of 5 mg/kg of RTD-1 and plasma levels were determined by LC-MS/MS and plotted versus time and number of injections (Methods). The plot shows the 1 h (n = 12) and 4 h (n = 11) plasma levels of mice receiving 1 injection i.p. at T = 0. All other values are plasma levels (mean +/− S.D.) obtained 1 h after additional daily i.p. doses administered for up to 6 days with times of collection by group: 25 h, n = 4; 73 h, n = 8; 121 h, n = 4; 145 h, n = 7.

To ascertain the systemic levels of RTD-1 found to be effective *in vivo*, plasma levels of RTD-1 were evaluated over a 24 h period after single 5 mg/kg i.v. or i.p. injections of BALB/c mice (Fig. 4a). A two-compartment model best described the i.v. data. The mean maximum concentration (Cmax) and time to reach the maximum concentration (Tmax) after i.p. injection were 0.67 µg/mL and 4 h, respectively. These data indicate that RTD-1 is slowly absorbed i.p. with 63.4% bioavailability (Suppl. Table 2). Goodness-of-fit plots for the final pharmacokinetic model are shown in Supplementary Fig. 1. RTD-1 plasma levels were also analyzed 60 or 240 min after additional daily 5 mg/kg i.p. doses administered up to 6 days. In each instance, RTD-1 levels were not statistically different from that obtained by a single dose, indicating that RTD-1 did not accumulate in plasma over the course of several injections (Fig. 4b). Of note, i.p. Cmax values were 5–25 fold lower than RTD-1 MICs against *C*. *albicans* (Table 1). Moreover, in 50% serum, the MIC for RTD-1 against *C*. *albicans* SC5314 was >100 µg/ml, indicating that antifungal efficacy of the peptide *in vivo* is not a direct antifungal effect.

### RTD-1 treatment promotes fungal clearance and modulates systemic inflammation in candidemic mice

**Figure 5:**
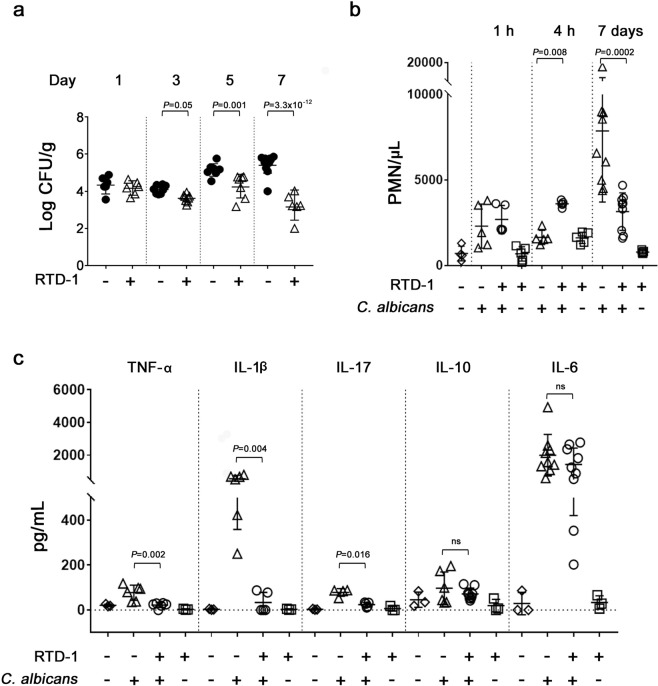
Effects of RTD-1 on host response to systemic candidiasis. (**a**) RTD-1 promotes clearance of *C*. *albicans*. Kidney fungal burdens were measured in mice infected with 3 × 10^5^ *C*. *albicans* SC5314 and treated i.p. at T = 0 with a single dose of 5 mg/kg RTD-1 or saline vehicle. Mice were euthanized 1, 3, 5 or 7 d after infection and CFU/g of kidney homogenate was determined by plating. Significance of RTD-1 treatment was analyzed by Fisher’s LSD test. (**b**) RTD-1 treatment reverses neutrophilia in candidemic mice. NaÏve or infected mice were treated with i.p. saline or a single dose of 5 mg/kg RTD-1 administered at the time of i.v. infection (as in Fig. 3a). Blood PMNs were quantified from individual animals 1 h, 4 h, and 7 days in each treatment group (n = 4 - 9 for each treatment cohort) and plotted as mean ± SD. RTD-1 treatment increased PMN levels at 4 h (*P* = 0.008) in infected mice, while significantly decreased PMNs (*P* = 0.002) 7 d after infection. (**c**) RTD-1 normalizes pro-inflammatory cytokines in candidemic mice. Mice (n = 3–9 for each cohort), treated as indicated, were euthanized 7 d after infection. Blood cytokines were quantified and are plotted as means ± SD, RTD-1 treatment effects were analyzed by Mann-Whitney test.

Fungal burdens in RTD-1-treated SC5314 candidemic mice were determined by quantifying CFU in kidney homogenates from animals treated i.p. once with 5 mg/kg of RTD-1 at T = 0. Fungal burden was quantified 1, 3, 5 and 7 days p.i. By day 3 p.i., a single RTD-1 treatment markedly reduced fungal burden compared with saline treated controls wherein fungal burden continued to increase during the 7 days observation period (Fig. 5a).

Neutrophils represent the first line of defense in invasive candidiasis, and susceptibility to systemic disease is markedly increased by persistent neutropenia^44,45^. Systemic disease is also associated with greatly elevated levels of proinflammatory cytokines^46^. While administration of RTD-1 to uninfected mice had little effect on blood cytokines or leukocytes, peptide treatment of candidemic mice significantly (*P* = 0.008) increased blood neutrophil counts 4 h p.i. (Fig. 5b). Interestingly, at this same time point, RTD-1 increased blood neutrophils 2.3 fold in naÏve mice (*P* = 0.32). However, by day 7, when saline treated candidemic mice had marked leukocytosis, neutrophil counts in the RTD-1 treated mouse cohort were significantly (*P* = 0.0002) lower, and essentially equivalent to that observed in this cohort at the 4 h time point (Fig. 5b). Consistent with these findings, RTD-1 treatment of candidemic mice resulted in a significant reduction of blood proinflammatory cytokines implicated in candida sepsis (TNF, IL-1β, and IL-17) 7 days p.i., while having no effect on IL-10 or IL-6 levels, both of which were elevated in candidemic mice (Fig. 5c). RTD-1 did not alter the levels of these cytokines in naÏve mice.

**Figure 6:**
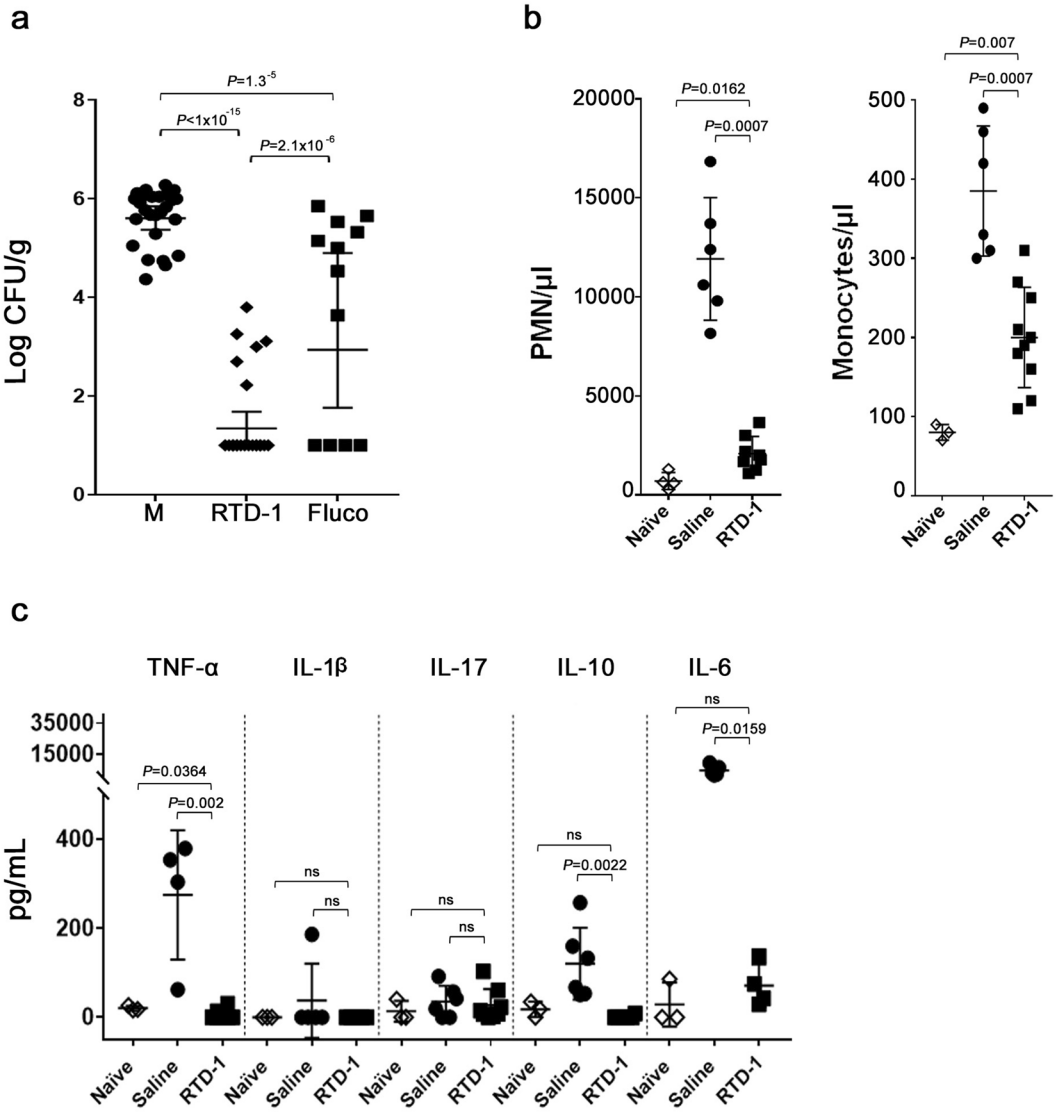
RTD-1 promotes fungal clearance and restores immune homeostasis in long term survivors. (**a**) RTD-1 mediated fungal clearance. Fungal burdens were quantified in homogenates of individual kidneys of long term survivors treated i.p. at T = 0 with saline, RTD-1 or Fluco (mice from studies presented in Fig. 3a,b). Saline-treated moribund mice (M) were euthanized between 5 and 10 d p.i. CFU/g (geometric means with 95% CI) were plotted for each treatment cohort and analyzed by Fisher’s LSD test. (**b**) RTD-1 normalizes blood neutrophils and monocytes in long term survivors of systemic candidiasis. Blood PMNs and monocytes were quantified in naÏve mice, moribund saline treated mice, and RTD-1 treated long term survivors (n = 6 per cohort; mice from studies presented in Fig. 3a,b). Effect of RTD-1 treatment was analyzed by Mann-Whitney test. (**c**) Cytokines normalized by RTD-1 in candidemic long term survivors. In long term survivors, RTD-1 treatment restored pro-inflammatory biomarkers TNF-α, IL-10, and IL-6 to levels equivalent to those of naÏve animals (n = 3-11 in each cohort). Effect of RTD-1 treatment was analyzed by Mann-Whitney test.

To assess the effect of RTD-1 treatment later in the disease course, we analyzed fungal burden, blood leukocytes, and inflammatory cytokines in RTD-1 treated, SC5314 infected mice at day 30 p.i. and in moribund, saline treated animals euthanized 5–10 p.i. (Fig. 3a,b). RTD-1 treatment markedly reduced kidney fungal burden (*P* < 1 × 10^−15^) and peptide treatment was substantially more effective than treatment with Fluco (*P* = 2.1 × 10^−6^; Fig. 6a). Of note, no culturable organisms were detected in 50% of the kidneys in RTD-1 treated mice. RTD-1-induced fungal clearance was accompanied by homeostatic normalization of circulating granulocytes which were markedly elevated in moribund saline-treated controls (Fig. 6b). In RTD-1 treated mice, levels of TNF, IL-10, and IL-6 were normalized to those of uninfected controls (Fig. 6c), complementing the homeostatic effects of RTD-1 on TNF, IL-1β, and IL-17 observed at 7 days p.i. (Fig. 5c).

### RTD-1 is efficacious in candidiasis mediated by MDR *C*. *albicans*

**Figure 7:**
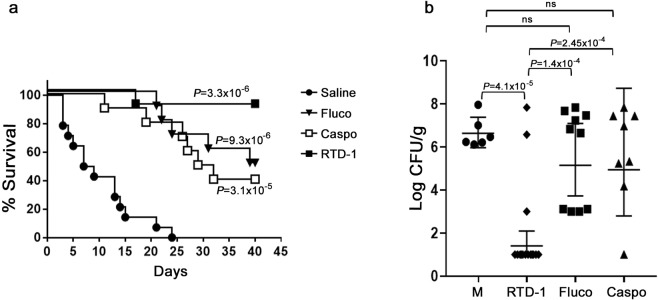
RTD-1 promotes survival and fungal clearance in systemic MDR candidiasis. (**a**) Efficacy of RTD-1 in *C*. *albicans* 53264 candidiasis. CD-1 mice infected i.v. with 1.8 × 10^7^ blastospores of *C*. *albicans* 53264 were treated i.p. with 5 mg/kg of RTD-1 (n = 10), Fluco (n = 10), Caspo (n = 10), or saline vehicle (n = 14) daily for 7 d beginning 24 h p.i. and monitored for 40 days. Log-rank test analysis demonstrated that each agent enhances survival compared to saline controls, with RTD-1 having the greatest efficacy (see Suppl. Table 1). (**b**) Clearance of *C*. *albicans* in long term survivors. Long term survivors (40 days) from each cohort in panel (**a**) were euthanized and kidney fungal burdens quantified, as were saline-treated moribund mice (M) euthanized between 5 and 24 d p.i. CFU/g (geometric means with 95% CI) were plotted for each treatment cohort and analyzed by Fisher’s LSD test.

To assess whether the effects of RTD-1 on drug sensitive *C*. *albicans* SC5314 extend to drug resistant isolates, CD-1 mice infected i.v. with Caspo-resistant *C*. *albicans* 53264 were treated i.p. with 5 mg/kg each of RTD-1, Caspo, or Fluco once per day for 7 days beginning 24 p.i. (as in Fig. 3d). CD-1, rather than BALB/c mice were used in the *C*. *albicans* 53264 challenge studies as this mouse strain was employed by Wiederhold *et al*. in the assessment of caspofungin efficacy against this pathogen^40^. As shown in Fig. 7A, each agent significantly (*P* < 0.0001) improved survival compared to saline controls, and RTD-1 was superior to Caspo (*P* = 0.029) but not Fluco (Fig. 7A). By survival endpoint analysis (χ2) at day 40 p.i, RTD-1 was superior to Fluco (*P* = 0.01) and Caspo (*P* = 0.001). Fungal burdens were determined in kidneys of long term survivors in the RTD-1, Caspo, and Fluco-treated cohorts (euthanized at day 40) and from moribund mice from the survival study in Fig. 7a. Of the agents tested, only RTD-1 significantly (*P* = 4.1 × 10^−5^) reduced fungal burden relative to moribund saline controls (Fig. 7b).

## Discussion

Several factors, including indiscriminate and overuse of antifungals, increasing use of immunosuppressive therapies, and nosocomial colonization of hospitalized patients has accelerated the emergence of MDR strains. The severity of this public health threat has gained worldwide attention. While the introduction of echinocandins, nearly two decades ago, provided a valuable alternative to azoles and polyene antibiotics^47,48^, MDR strains resistant to echinocandins are diagnosed with increasing frequency^40,49^ highlighting the need for new therapeutic approaches. Studies presented here suggest that RTD-1, a macrocyclic peptide expressed in leukocytes of rhesus macaques, may represent a new therapeutic modality based on its efficacy in systemic candidiasis, including disease mediated by a caspofungin-resistant MDR strain of *C*. *albicans*.

RTD-1 is the prototype θ-defensin, the only known backbone cyclized polypeptides in the Animal Kingdom. All natural θ-defensin isoforms are 18-amino acid macrocycles stabilized by a six cysteine, tridisulfide core. Like other host defense peptides (HDPs), θ-defensins were discovered by screening for antimicrobial activities in lysates of rhesus macaque leukocytes which revealed that RTD-1 and closely related isoforms (RTDs 2–5) were broadly bactericidal and fungicidal *in vitro* against *C*. *albicans* and *Cryptococcus neoformans*^31–33,50^. As reported here and elsewhere^39^, RTD-1 kills *Candida* spp. *in vitro* by mechanisms that are temporally linked to cell death. For example, RTD-1 rapidly (<10 min) permeabilized *C*. *albicans* to propidium iodide, induced ATP release and reactive oxygen species in planktonic cells^39^. Moreover, θ-defensins are fungicidal against MDR *C*. *albicans* and non *albicans Candida* spp.^39^.

Here we report for the first time that RTD-1 is effective against both planktonic cells and biofilms of drug sensitive and MDR *C*. *albicans in vitro*. However, MICs of RTD-1 were 10–20 higher than Cmax plasma levels obtained following single or multiple dosing regimens in mice. Further, in the presence of 50% mouse serum, MICs were at least 100 fold higher than plasma Cmax values. Therefore, it is highly unlikely that the efficacy of RTD-1 in mouse systemic candidiasis is a direct antifungal effect, but rather is mediated by host-directed mechanisms, producing marked survival enhancement that was superior to that obtained with Fluco, and accompanied by efficient fungal clearance. Consistent with host-directed mechanisms, RTD-1 was highly effective in systemic candidiasis against drug sensitive and MDR strains of *C*. *albicans*.

While the pharmacologic basis of RTD-1 efficacy in candidemia is yet to be determined, data presented here suggest that peptide administration may promote the mobilization of neutrophils, as blood levels of these cells were significantly elevated in RTD-1 treated candidemic mice (Fig. 5), and we speculate that this early phase neutrophilia contributes to the reduction in systemic fungal burden detected as early as 3 days after a single treatment with RTD-1. As noted above, neutrophils play an important role in host defense against *C*. *albicans* infection via phagocytosis and formation of neutrophil extracellular traps^51,52^.

RTD-1 treatment of candidemic mice also had a profound effect on inflammatory cytokines over the course of infection. By day 7 p.i., RTD-1 treated mice (single dose at T = 0) had marked reductions in TNF, IL-1β, and IL-17 compared to saline treated animals. Additionally, in later stages of the disease course, RTD-1 treatment markedly prolonged survival and in these mice, levels of IL-10 and IL-6 normalized compared to moribund saline-treated controls. This was accompanied by significant reductions in blood neutrophils and monocytes. Thus, RTD-1 treatment of candidemic mice induced transient neutrophilia, followed by profound reductions in fungal burden compared to saline controls (*P* < 1 × 10^−15^), and in this regard, RTD-1 treatment was markedly superior to Fluco (Fig. 6). In parallel, peptide treatment homeostatically normalized granulocytes and inflammatory cytokines. The effects of  RTD-1 in systemic candidiasis are consistent with results of studies in which the peptide was analyzed in models of *E*. *coli* and polymicrobial sepsis^35^, *P*. *aeruginosa* pneumonia^37,53^, and in murine SARS Coronavirus pneumonitis^36^. In each of these studies, RTD-1 therapeutically modified the course of disease and efficacy was associated with modulation of pathologic inflammation.

The therapeutic and pharmacologic properties of RTD-1 in systemic candidiasis suggest that the peptide modifies the host damage-response framework articulated by Casadevall^54^. Consistent with this model, RTD-1 treatment stimulates neutrophilia early in infection but suppresses inflammatory cytokines and promotes homeostatic restoration of blood granulocyte levels and proinflammatory cytokines which are chronically elevated in saline treated candidemic mice. Also consistent with this model is the finding that the dose-effect relationship of RTD-1 is an inverted “U”, as shown by the increasing efficacy as dose levels are increased up to 5 mg/kg, but a marked decrease in efficacy with dosing at 10 and 20 mg/kg (Fig. 3b,e). Of note, i.p. doses of at least 50 mg/kg RTD-1 were well tolerated in uninfected mice (Results), suggesting that higher levels of the peptide are less well tolerated in candidemic mice or that elevated concentrations are overly immunosuppressive. Further studies are required to delineate PK/pharmacodynamic relationships underlying the inverted U effects of RTD-1 treatment of systemic candidiasis.

Despite the remarkable *in vitro* properties of HDPs, efforts to develop them as therapeutics have met with limited success^55^. Barriers encountered include lack of peptide stability, toxicity, adverse proinflammatory properties, and poor bioavailability. In the context of antifungal therapeutics, we are aware of only two reports that demonstrate peptide efficacy in systemic candidiasis. In the first, Tavares *et al*. used plant defensin RsAFP2y in a prophylaxis model to show that that i.v. administration of 14 mg/kg of this peptide, given 1 h prior to i.v. infection with *C*. *albicans* strain 78, reduced fungal burden 5 d after infection, but no impact on survival was evaluated for efficacy in a mouse candidiasis model^56^. More recently, IDR-1018, an analog of bovine bactenecin 2 A, was evaluated in a mouse candidiasis model^57^. Daily intraperitoneal administration of 10 mg/kg of IDR-1018 beginning 24 h after retroorbital infection with a clinical isolate of *C*. *albicans* modestly improved survival up to 8 days p.i. which was accompanied by similarly modest reduction in renal fungal burden^57^. Of note, several small non-peptidic HDP-mimics, designed by Scott, Diamond and colleagues, were active *in vitro* against several Fluco-resistant *Candida* spp., and some were effective in *C*. *albicans* SC5314 systemic candidiasis^58,59^.

Our study is the first to show therapeutic efficacy, e.g., long term survival accompanied by fungal clearance, of a naturally occurring peptide in systemic candidiasis. Findings presented here demonstrate that RTD-1 both promotes fungal clearance and suppresses pathologic inflammation, and these effects augment host responses to infection by drug sensitive and MDR *C*. *albicans* strains. Of note, RTD-1 is highly stable in serum and plasma^35^, to host^60^ and fungal proteases^39^, is non-immunogenic^61^, and non-toxic (Results). Thus, RTD-1 provides a template for design of bioinspired macrocyclic compounds that may contribute an unmet need for new antifungal therapies.

## Methods

### Ethics

All methods methods were performed in accordance with relevant federal, state, and institutional guidelines. Animal use protocols were approved by The University of Southern California (USC) Institutional Animal Care and Use Committee (IACUC), Protocol #20538.

### C. albicans strains

Reference strain *C*. *albicans* SC5314 was obtained from American Type Culture Collection. Caspofungin resistant clinical isolates of *C*. *albicans* 43001 and 53264 were kindly provided by Dr. Nathan Wiederhold (Fungus Testing Laboratory at the University of Texas Health Science Center at San Antonio, TX).

### Peptides and antifungal drugs

Highly pure (>98%) RTD-1 hydrochloride was produced by solid phase peptide synthesis as described^31,32^. Caspofungin and fluconazole were purchased from Sigma-Aldrich (St. Louis, MO). RTD-1 was dissolved at 0.5 mg/ml in 0.01% acetic acid and antifungal drugs were prepared in sterile water. For animal injection, peptides and antifungals drugs were diluted in filter sterilized saline solution.

### *In vitro* antifungal assays

*C*. *albicans* strains were tested against RTD-1, fluconazole, and caspofungin in microdilution broth in accordance with Clinical and Laboratory Standards Institute (CLSI) document M27-A2 with the exception that 25 mM MOPS was used in place of 165 mM MOPS, as described previously^39^. Overnight cultures were harvested by centrifugation, washed twice with Ca^2+^ and Mg^2+^-free PBS, pH 7.2, and suspended in test medium (RPMI 1640 medium, 25 mM MOPS, 2 mM L-glutamine) at 10^4^ CFU/ml^62^. Incubations were conducted in flat bottom, 96-well microtiter plates (Greiner bio-one, Monroe, NC) containing 0.2 ml test medium per well. Duplicate 0.1 ml aliquots of serial two-fold dilutions of peptides and antifungal drugs were dispensed into wells, each of which was inoculated with 0.1 ml of fungal cell suspension containing 1 × 10^3^ CFU. Plates were incubated at 30 °C for 48 hours after which A_600_ was determined using a SpectraMax M5e plate reader. Minimal inhibitory concentration (MIC) was defined as the lowest agent concentration that completely inhibited growth as determined by A_600_ absorbance.

Minimal fungicidal concentration (MFC) was determined by plating, on YPD plates, 10 µl from wells in MIC assay without cell growth and the first well with measurable turbidity as described^63–66^ and corresponding aliquots from the no-agent controls. Under MIC incubation conditions, no filamentation occurred allowing for accurate CFU counts after plating on YPD plates which were incubated for 24 h at 30 °C. MFC is reported as the lowest concentration of peptide or antifungal drug that killed ≥99% of the input organisms.

MICs and MFCs of RTD-1 were also performed in the presence of 50% mouse serum. Serum was prepared from blood collected by cardiac puncture from BALB/c mice. After coagulation at room temperature, serum was collected by centrifugation (200 g for 10 min followed by 23,000 g for 15 min), and diluted with an equal volume of yeast inoculum in MIC test medium described above.

Kinetics of RTD-1 candidacidal activity was determined at peptide concentrations ranging from 0 to 10 µg/ml using a liquid suspension assay described previously^39^. Overnight cultures of *C*. *albicans* 53264 or 43001 were grown to mid-log phase in 5% yeast peptone dextrose broth (YPD) (Difco) at 30 °C. Approximately 2 × 10^6^ CFU/ml were incubated with 0, 0.3, 1, 3 and 10 µg/ml RTD-1 or 10 µg/ml caspofungin, suspended in 10 mM PIPES, pH 7.4, plus 5 mM glucose (PG), in 96-well plates with shaking at 60 RPM at 37 °C. At timed intervals, samples were diluted 1:1000 fold into PG buffer and aliquots plated onto YPD agar plates (TekNova), and surviving organisms quantified by colony counting after incubation for 24 h at 30 °C.

Fungicidal activities of RTD-1 and caspofungin were similarly determined in whole blood. Citrate anticoagulated blood was obtained by cardiac puncture from CD-1 mice. Approximately 5 × 10^4^ CFU/ml of C. albicans SC5314 or 53264 were incubated with 0, 1, 3, 10, 30 and 100 µg/ml RTD-1 or caspofungin in 96-well plates with whole blood (85% vol/vol final) with shaking at 60 RPM at 37 °C. After 2 h, samples were diluted 1:10 into 10 mM PIPES, pH 7.4 containing 0.05% YPD and aliquots were plated onto YPD agar plates (Teknova) and CFU were quantified as above.

### Biofilm studies

The antifungal activities of RTD-1, caspofungin and fluconazole were evaluated against biofilms of *C*. *albicans* SC5314 and 53264 essentially as described by Pierce *et al*.^67^. Briefly, biofilms established for 0 h (preadhesion), 2 h (adherent), or 24 h (mature) were developed by adding 1 × 10^5^ yeast cells in 50 or 100 µl of RPMI-1640 to wells of flat-bottom 96-well microtiter plates (Costar, Corning, NY). Fifty or 100 µl aliquots of serial 2-fold dilutions of peptide or antifungal drugs were added to wells in triplicate and incubated for 24 h or 48 h prior to analysis of biofilm metabolic activities. Concentrations of reagents used were as follows: RTD-1 - 0.09 – 100 µg/ml (0.05–48 µM), Fluco – 1–250 µg/ml (3.3–816.3 µM); Caspo- 0.003–8 µg/ml (0.0003–7.3 µM). Analysis of the effect of each agent on *Candida* biofilms was performed using an XTT colorimetric assay^67^. One hundred µl of 1 µM XTT/menadione added to each biofilm well, plates were incubated in the dark for 3 h at 37 °C, and 80 µl of supernatant was transferred to a new 96-well flat-bottom microtiter plate (Greiner Bio-one, Monroe, NC). Absorbance was measured at 490 nm on a SpectraMax^®^ i3x (Molecular Devices). Readings were plotted as average of triplicates after subtracting the corresponding values for negative controls (XTT/menadione only).

### Systemic candidiasis model

BALB/c female mice (7–8 weeks old) and CD-1 female mice (4–5 weeks old) were obtained from Charles River Laboratories and allocated randomly in groups of five mice per cage. Mice were maintained on a 12 h light/dark cycle in thermostatically controlled rooms for the duration of the experiment. *C*. *albicans* inocula were prepared fresh each day from saturated cultures grown overnight in YPD medium, washed in sterile PBS, and fungi were counted with hemocytometer. Stock suspensions were prepared in sterile PBS. BALB/c mice were challenged with 2 × 10^6^ CFU/ml wild-type *C*. *albicans* reference strain SC5314, while CD-1 mice were challenged with 1.8 × 10^7^ CFU/ml (0.15–0.2 ml) *C*. *albicans* clinical isolate 53264 by i.v. injection into the lateral caudal tail vein using a 28 G 1/2 inch needle. Animals were treated by i.v., s.c., or i.p. routes immediately post-infection (p.i.) or 24 h p.i. with sterile saline (control), RTD-1, fluconazole, or caspofungin administered at the concentrations indicated in a 0.200 ml. Mice were weighted and monitored daily for general clinical condition. Mice were euthanized as approved by the USC IACUC and in compliance with the National Institutes of Health Guide for the Care and Use of Laboratory Animals. All experiments were repeated at least three times.

To assess systemic fungal clearance, renal fungal burden was determined. Mice were euthanized at times indicated, and both kidneys were weighed, homogenized in 5 ml of sterile PBS, and serial dilutions of homogenate were plated on YPD agar plates and incubated at 30 °C for 2 days. CFU per gram of kidney was calculated.

### RTD-1 pharmacokinetics (PK)

Single dose PK of RTD-1 was evaluated by quantifying plasma peptide concentrations at intervals following 5 mg/kg bolus injections administered i.v. and i.p. to CD-1 mice. At indicated intervals, plasma was prepared from EDTA-anticoagulated blood collected aseptically by terminal cardiac puncture. Plasma was diluted 1:10 or 1:20 into 5% formic/5% acetonitrile. RTD-1 plasma concentrations were determined by reverse-phase liquid chromatography (XBridge phenyl 3.5 µm column, Waters) on an Acquity H-Class UPLC (Waters) with tandem electrospray mass spectroscopy on a Xevo TQ-S running MassLynx V4.1 (Waters). Quantitative mass spectroscopy was performed by multiple-reaction monitoring transition 417.38 > 517.14, with area under the curve determined by TargetLynx (Waters). Pharmacokinetic modeling of intravenous RTD-1 concentration-time data was performed with ADAPT (version 5) software using the naïve pooled data approach. One- and two-compartment models were evaluated. Model selection was based on the Akaike information criterion (AIC) and the Bayesian information criterion (BIC) scores, the likelihood ratio test (LRT), and goodness-of-fit plots. Concentration data below the lower limit of quantitation were treated as missing for pharmacokinetic analysis. Area under the curve (AUC_24_) was calculated by taking the average of RTD-1 concentrations at each time point and applying the linear trapezoidal method. Bioavailability (F) for i.p. injection was determined using the ratio of AUCs from i.p. and i.v.

### Hematology and cytokine analyses

EDTA-anticoagulated blood was collected aseptically by terminal cardiac puncture and analyzed for complete blood cell count using a HemaVet 950 FS hematology analyzer (Drew Scientific). Plasma cytokine levels plasma were quantified using a mouse-specific MILLIPLEX MAP kit (Millipore) as described^35^.

### Statistical analyses

Survival curves were compared using the log rank (Mantel-Cox) test. Blood levels of PMNs and monocytes, and plasma levels of cytokines were compared using Mann-Whitney test. One-way ANOVA was used for analysis of variance of fungal burden data, followed by uncorrected Fisher’s Least Significant Difference (LSD) test. Significance of survival endpoint was calculation by χ^2^ analysis (Fig. 7 only). All statistical analyses employed GraphPad Prism 8.12.

## Supplementary information

Supplementary information
