# Supplementary material for: Rhesus Theta Defensin 1 Promotes Long Term Survival in Systemic Candidiasis by Host Directed Mechanisms
Source: Sci Rep. 2019 Nov 15;9:16905. doi: 10.1038/s41598-019-53402-z (PMC6858451; doi:10.1038/s41598-019-53402-z)
Supplement: Supplementary file 1 — Supplementary information [file 41598_2019_53402_MOESM1_ESM.pdf]

Rhesus Theta Defensin 1 Promotes Long Term Survival in Systemic Candidiasis by Host Directed Mechanisms

Virginia Basso[1], Dat Q. Tran[1,2], Justin B. Schaal[1], Patti Tran[1], Yoshihiro Eriguchi[1], Diana Ngole[1], Anthony E. Cabebe[1], A young Park[3], Paul M. Beringer[3], André J. Ouellette[1,4], and Michael E. Selsted*[1,2,4]

[1]Department of Pathology and Laboratory Medicine, Keck School of Medicine, University of Southern California, Los Angeles, California, United States of America

[2]Oryn Therapeutics, Vacaville, California, United States of America

[3]Department of Clinical Pharmacy, School of Pharmacy, University of Southern California, Los Angeles, United States of America

[4]Norris Comprehensive Cancer Center of the University of Southern California, Los Angeles California, United States of America

*Address correspondence to Michael E. Selsted, selsted@med.usc.edu

Suppl. Fig. 1

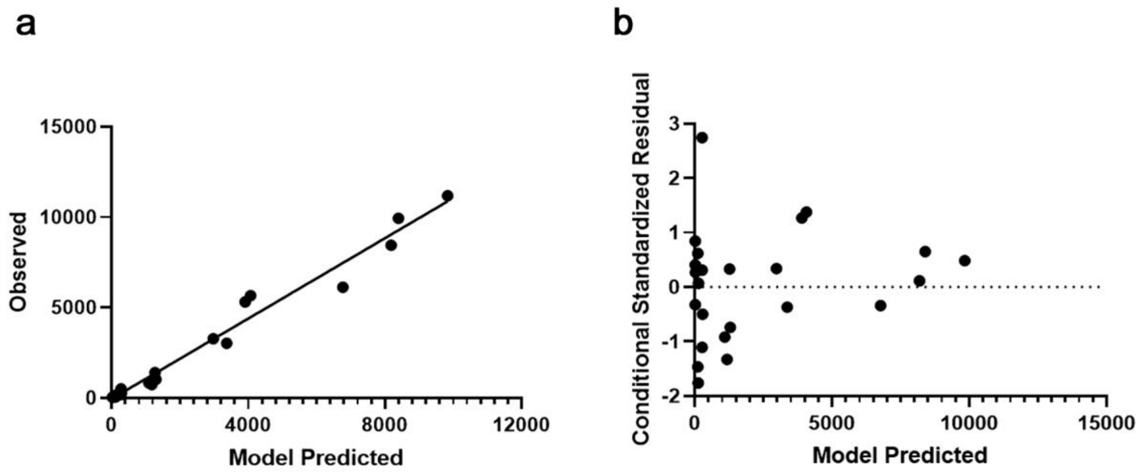

Suppl. Figure 1. Goodness-of-fit plots of the final PK model. (a) Observed RTD-1 concentrations versus model predicted concentrations following i.v. injection (R^2 = 0.9774; slope ± SE = 1.111-61.04) using linear regression, and **(b)** conditional standardized residuals versus model prediction.

Suppl. Table 1. Log-rank analysis for Fig. 3.

	RTD-1 i.p.	RTD-1 s.c.	RTD-1 i.v.	Caspo	Fluco
**Saline**	3.3×10^{-7}	1.5×10^{-5}	6.9×10^{-4}	4.3×10^{-7}	4.3×10^{-7}
**RTD-1 i.p.**	–	ns	ns	0.019	ns
**RTD-1 s.c.**	–	–	ns	3.1×10^{-3}	ns
**RTD-1 i.v.**	–	–	–	5.7×10^{-3}	ns
**Caspo**	–	–	–	–	0.014

a. RTD-1 efficacy is independent of route of peptide administration.

b. RTD-1 efficacy *in vivo* is dose-dependent.

	20 mg/kg	10 mg/kg	5 mg/kg	2.5 mg/kg	0.5 mg/kg	0.25 mg/kg
**Saline**	6.3×10^{-5}	0.013	3.3×10^{-7}	1.5×10^{-6}	1.5×10^{-4}	4.0×10^{-5}
**20 mg/kg**	-	6.3×10^{-5}	3.7×10^{-5}	6.3×10^{-5}	6.3×10^{-5}	6.3×10^{-5}
**10 mg/kg**	–	–	9.4×10^{-5}	1.2×10^{-3}	2.1×10^{-3}	0.024
**5 mg/kg**	–	–	–	ns	ns	ns
**2.5 mg/kg**	–	–	–	–	ns	ns
**0.5 mg/kg**	–	–	–	–	–	ns

c. Dependence of single dose RTD-1 efficacy on time of administration.

	RTD-1 T=0 h	RTD-1 T=1 h	RTD-1 T=3 h	RTD-1 T=6 h
**Saline**	3.3×10^{-7}	2.7×10^{-3}	1.5×10^{-4}	1.7×10^{-3}
**RTD-1 T=0 h**	–	4.3×10^{-3}	2.5×10^{-4}	7.0×10^{-3}
**RTD-1 T=1 h**	–	–	ns	ns
**RTD-1 T=3 h**	–	–	–	ns

d. Daily RTD-1 treatment beginning 24 h after infection promotes survival.

	RTD-1	Caspo	Fluco
**Saline**	3.4×10^{-6}	3.4×10^{-6}	3.4×10^{-6}
**RTD-1**	–	ns	ns
**Caspo**	–	–	4.7×10^{-3}

Supplementary Table 2. Parameter estimates for the final pharmacokinetic model for RTD-1

	Final model	
**Model parameter**	**Mean**	**SE (CV%)**
CLt (mL/h)	14.17	7.179
Vc (mL)	14.58	13.19
CLd (mL/h)	3.248	18.22
Vp (mL)	24.88	19.02
K10 (h^{-1})	0.9719	8.379
λ1 (h^{-1})	1.221	8.829
λ2 (h^{-1})	0.1039	13.20
T1/2-λ1 (h)	0.5676	8.829
T1/2-λ2 (h)	6.672	13.20
F	0.6344	

CLt, total clearance; Vc, central volume of distribution; Ka, absorption rate constant; CLd, distributional clearance; Vp, peripheral volume; K10, elimination rate constant from the central compartment; λ1, distribution rate constant, λ2, terminal rate constant; T1/2-λ1, distribution half-life; T1/2-λ2, terminal half-life; F, bioavailability.
